# Association between NSAID and Statin Therapy and the Incidence of Intravitreal Anti-vascular Endothelial Growth Factor Injections and Nd:YAG Laser Treatment after Cataract Surgery in Finland

**DOI:** 10.18502/jovr.v17i2.10789

**Published:** 2022-04-29

**Authors:** Sirpa Loukovaara, JJari Haukka

**Affiliations:** ^1^Unit of Vitreoretinal Surgery, Department of Ophthalmology, Helsinki University Hospital, and Individualized Drug Therapy Research Program, University of Helsinki, Helsinki, Finland; ^2^Department of Public Health, University of Helsinki, Helsinki, Finland; ^3^Faculty of Medicine and Health Technology, Tampere University, Finland

**Keywords:** Anti-inflammatory, Capsulotomy, Cataract Surgery, Cystic Macular Edema, Epidemiology, Intravitreal Injection, Ketorolac, Nationwide Cohort Study, NSAID; Secondary Cataract, Statin Therapy

## Abstract

**Purpose:**

To examine the association between the use of topical non-steroidal anti-inflammatory (NSAID) medication, systemic statin therapy, and the incidence rate of two of the most common postsurgical procedures in adult patients undergoing cataract surgery in Finland between January 1, 2010 and December 31, 2016.

**Methods:**

This retrospective, nationwide cohort study considered 176,052 cataract operations coded with the International Classification of Disease coding: early adult (H25.0), normal (H25.1), other senile (H25.8), pre-senile (H26.02), or other (related to trauma, other eye disease, or medication). Operations were linked to purchased and reimbursed medications using Anatomical Therapeutic Chemical codes. The incidence rate of intravitreal anti-vascular endothelial growth factor (VEGF) injections, and neodymium-doped yttrium aluminum (Nd:YAG) laser treatments of posterior capsular opacification were evaluated using the Poisson regression model.

**Results:**

In our registry cohort, patients with a prescription of topical NSAID (ketorolac) at the time of cataract surgery were less likely treated with intravitreal anti-VEGF injections after surgery (adjusted Poisson regression model IRR 0.3; 95% CI: 0.15–0.60, *P* = 0.0007), and also had reduced incidence of Nd:YAG laser (0.59, CI: 0.43–0.81, *P* = 0.0011) treatments. Unlike topical NSAID, the use of systemic statin therapy was not associated with these two most common surgical procedures (RR 1.04, 95% CI: 0.96–1.12, *P* = 0.33).

**Conclusion:**

The use of topical NSAIDs is associated with reduced rates of intravitreal anti-VEGF injections and Nd:YAG laser treatments after cataract surgery. More observational and experimental studies are warranted to confirm possible benefits of topical NSAID administration after cataract surgery.

##  INTRODUCTION

Cataract surgery is the most commonly performed surgical procedure in the world.^[1]^ Posterior capsular opacification (PCO) can occur a few months to many years after uncomplicated clear corneal phacoemulsification with intraocular lens implantation, demanding further treatment with neodymium-doped yttrium aluminum (Nd:YAG)-laser capsulotomy.^[2]^ In some operated eyes, removal of cataract can also cause postoperative cystoid macular edema (CME), leading to the need for intravitreal anti-vascular endothelial growth factor (VEGF) treatment.^[3,4]^

To treat postoperative inflammation after cataract surgery, both topical steroidal and non-steroidal anti-inflammatory (NSAID) drops are widely used. Lately, immunomodulatory systemic statin therapy has also been shown to be beneﬁcial in ophthalmology.^[5,6,7]^ The use of systemic statins are known to decrease inﬂammatory responses, reduce ﬁbrosis formation, as well as lower the risk of ophthalmic surgical interventions.^[6,8,9,10,11,12]^ Recently, a systematic review and meta-analysis of a sample of approximately 310,000 patients showed that the use of statins could moderately increase the risk of future cataracts (relative risk, 1.13; 95% CI: 1.01–1.25).^[13]^ However, more information is needed to analyze the potential association of systemic statin use, and the rate of intravitreal anti-VEGF injection treatment or Nd:YAG-laser treatment after cataract surgery.

The purpose of this study was to examine the incidence rate of intravitreal anti-VEGF injections and Nd:YAG laser procedures after cataract surgery in adult patients in a large Finnish cohort in addition to establishing whether the rate of administering these two most common procedures correlates with the use of topical NSAID drops and/or systemic statin therapy at the time of surgery?

##  METHODS

This was a population-based epidemiologic study of adult patients who underwent cataract surgery from July 1, 2010 to December 31, 2016 in Finland.

In brief, we obtained information of cataract operations (phacoemulsification with implantation of artificial lens in posterior chamber; coded as CJE20) based on the Nordic Medico-Statistical Committee (NOMESCO) codes, and data were retrieved from the database of the National Institute for Health and Welfare (THL) (permission ref. THL/2038/5.05.00/2017).

The adult cataract eyes were coded with International Classification of Disease (ICD)-codes as follows: H25.0 (early adult cataract), H25.1 (normal cataract), H25.8 (other senile cataract), H26.02 (presenile cataract), or other (i.e., related to trauma, other eye disease, or medication).

The data consisted of the patient-related variables (date, type of operation/procedure code, laterality of the eye, age, sex, systemic comorbidities).

After cataract surgery, the two major ophthalmic surgical procedure codes defined by NOMESCO were included: intravitreal injection of anti-VEGF medication (CKD05) and treatment of PCO with neodymium-doped yttrium aluminum (Nd:YAG) laser (CJB10).

All purchased and reimbursed medications were recorded in the Finnish Prescription Register with Anatomical Therapeutic Chemical (ATC) code (WHOCC - ATC/DDD Index. 2012). For each drug, reimbursement-related factors, including the dispensing date (date of purchase), ATC code, and the quantity dispensed (amount in defined daily doses) were recorded.

We included the following drugs (purchased during the prior six months) as time-dependent variables: statins (ATC code C10AA; simvastatin, atorvastatin, rosuvastatin, other), insulins (A10A), and other diabetes drugs (ODD) (A10B). Patients were classed as statin users if they had purchased the prescription for any statins in the six months prior to the cataract surgery. Patients were prescribed different statin doses, once or twice daily, but this could not be taken into account in the analysis.

We analyzed the data of ophthalmic drugs with ATC codes starting with code S (covering steroids, NSAIDs, antibiotic, glaucoma, anti-allergic, immunomodulatory, and lubricating drops). These drugs were prescribed one month before or one week after the cataract operation [Suppl Table 1]. Medications-related data were obtained from the Social Insurance Institution of Finland (KELA Permission ref. 93/522/2017). The frequency of topically administered NSAIDs could not be taken into account in the final analysis.

Finnish Registry for Reimbursed Medication includes reimbursement rights and diagnosis, and special reimbursement rights. We used the following reimbursement rights (no/yes) as covariates: Diabetes, Hypothyreosis, Psychiatric and other mental illnesses, Glaucoma, Breast cancer, Prostate cancer, Transplantation, Other cancer, Kidney disease with dialysis, Chronic heart disease, Connective tissue disease, Rheumatic diseases and other related conditions, Chronic hypertension, Coronary heart disease and hypercholesterolemia, Chronic arrhythmia, Colitis ulcerosa, and Crohn´s disease.

The dates and cause of death (ICD-10) were obtained from Statistics Finland (TK-52-1785-18). All data from four registers were linked by means of the unique personal identification number assigned to all people living in Finland.

The follow-up started at first procedure of any type of cataract operation type and ended on death or December 31, 2016. The two most common secondary procedures, that is, anti-VEGF injection or Nd:YAG laser treatment between the first operation and the end of follow-up (death or December 31, 2016) were recorded.

The ethics approval was obtained from the institutional committees of the Hjelt Institute, University of Helsinki, and the Hospital District of Helsinki and Uusimaa, Helsinki, Finland. The study was register-based and without patient contact.

### Statistical Analysis

Intravitreal anti-VEGF injection and Nd:YAG-laser treatments were the main endpoints, and the completion of the follow-up process or death were treated as censoring events. The main exposures were topical NSAID (ketorolac) and systemic immunomodulatory statin treatment defined by having prescriptions both before and after operations in half-year time windows. Current diabetes medication (insulin, other diabetes drugs [OAD]) were defined in a similar way.

We modelled incidence of secondary operation, that is, need for the anti-VEGF and Nd:YAG-laser treatment using Poisson regression models. Incidence rate ratios (IRR) which were calculated along with the cumulated follow-up time were taken into account. Confounding was controlled using background variables (sex, age, baseline usage of insulin, and other diabetes drugs [OAD], chronic diseases, calendar year of cataract surgery, and time since start of follow-up at the time of cataract surgery). We used natural splines with knots in 0.5, 1.5, 2.5, 3.5, 4.5, and 5.5 years for modelling time from the start of the follow-up period. We calculated predicted incidence as a function of time from the start of the follow-up period using the lowest category for each covariate. All calculations were carried out using R language.^[14]^

**Figure 1:**
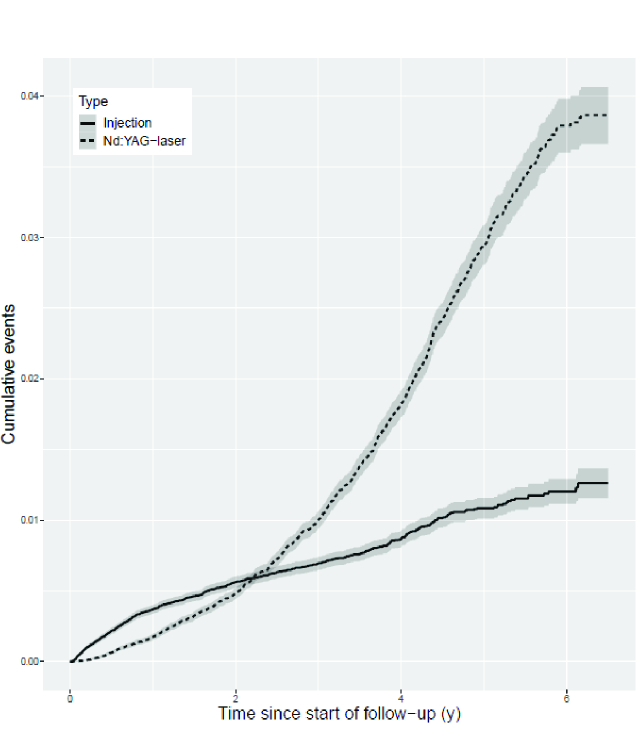
Kaplan-Meier cumulative events curves with 95% confidence intervals.

**Figure 2:**
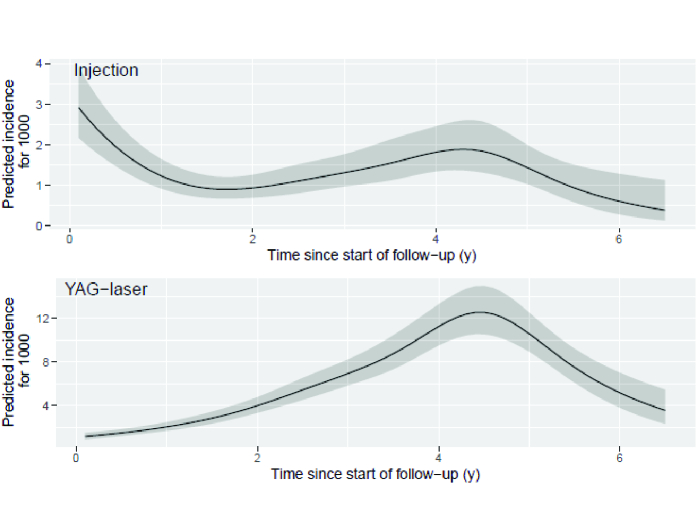
Predicted incidence with 95% confidence interval for intravitreal anti-VEGF injection and Nd:YAG-laser capsulotomy based on Poisson regression model.

**Table 1:** Basic characteristics of Finnish study cohort (n = 176 052).

**ICD-10 code**	**Type of cataract **
	**H25.0**	**H25.1**	**H25.8**	**H26.02**	**Other**	**Overall**
	**Early adult**	**Normal adult**	**Other senile**	**Pre-senile**		
*N*	* *	6432	138765	19738	3640	7473	176052
Sex, female (%)		4048 (62.9)	87685 (63.2)	12345 (62.5)	1949 (53.5)	4307 (57.6)	110334 (62.7)
Age (median [IQR])		75.97 [69.18, 81.37]	75.91 [69.68, 81.19]	76.73 [70.20, 81.95]	55.38 [49.90, 59.46]	69.88 [59.56, 77.59]	75.58 [68.91, 81.05]
Age (%; yr)	45	12 (0.2)	598 (0.4)	32 (0.2)	372 (10.2)	567 (7.6)	1581 (0.9)
	45–65	912 (14.2)	16336 (11.8)	2105 (10.7)	3124 (85.8)	2137 (28.6)	24614 (14.0)
	>65	5508 (85.6)	121831 (87.8)	17601 (89.2)	144 (4.0)	4769 (63.8)	149853 (85.1)
Insulin (%)		388 (6.0)	9884 (7.1)	1741 (8.8)	307 (8.4)	768 (10.3)	13088 (7.4)
Oral antidiabetic medication (%)		980 (15.2)	22845 (16.5)	3450 (17.5)	394 (10.8)	1048 (14.0)	28717 (16.3)
Simvastatin (%)		1439 (22.4)	29770 (21.5)	4186 (21.2)	389 (10.7)	1229 (16.4)	37013 (21.0)
Atorvastatin (%)		469 (7.3)	12264 (8.8)	1797 (9.1)	169 (4.6)	541 (7.2)	15240 (8.7)
Rosuvastatin (%)		153 (2.4)	4005 (2.9)	554 (2.8)	69 (1.9)	160 (2.1)	4941 (2.8)
Other statin (%)		157 (2.4)	3776 (2.7)	596 (3.0)	26 (0.7)	162 (2.2)	4717 (2.7)
Any statin (%)		2216 (34.5)	49783 (35.9)	7129 (36.1)	651 (17.9)	2088 (27.9)	61867 (35.1)
Ketorolac (%)		44 (0.7)	1985 (1.4)	1080 (5.5)	90 (2.5)	261 (3.5)	3460 (2.0)
**Comorbidities**	Glaucoma (%)	546 (8.5)	12112 (8.7)	1303 (6.6)	151 (4.1)	1523 (20.4)	15635 (8.9)
	Diabetes (%)	799 (12.4)	19930 (14.4)	3100 (15.7)	381 (10.5)	964 (12.9)	25174 (14.3)
	Connective tissue diseases, rheumatoid arthritis, and comparable diseases (%)	199 (3.1)	3684 (2.7)	556 (2.8)	94 (2.6)	255 (3.4)	4788 (2.7)
	Chronic coronary heart disease (%)	558 (8.7)	14107 (10.2)	2255 (11.4)	133 (3.7)	561 (7.5)	17614 (10.0)

The eyes with cataract were coded with International Classification of Disease (ICD)-codes as follows: H25.0 (early adult cataract), H25.1 (normal cataract), H25.8 (other senile cataract), H26.02 (presenile cataract), or other (i.e., related trauma, other eye disease, or medication). OAD, oral antidiabetic drug; IQR, interquartile range.

**Table 2:** Incidence rates (per 100 person-years) for Nd:YAG laser capsulotomy and intravitreal anti-VEGF injection with 95% confidence interval. Incidence rate ratio (IRR) from univariate Poisson model with 95% confidence interval.

**Group**	**Person-years (1/100)**	N	**Rate (1/100)**	**IRR**	N	**Rate (1/100)**	**IRR**
		**YAG-laser treatment**	**Injection**
Sex	Male	1771	593	0.33 (0.31–0.36)	(reference)	504	0.28 (0.26–0.31)	(reference)
	Female	3172	1675	0.53 (0.50–0.55)	1.58 (1.44–1.73)	676	0.21 (0.20–0.23)	0.75 (0.67–0.84)
Age (yr)	<45	48	67	1.39 (1.08–1.76)	(reference)	31	0.64 (0.44–0.91)	(reference)
	45–65	759	467	0.62 (0.56–0.67)	0.44 (0.34–0.57)	164	0.22 (0.18–0.25)	0.34 (0.23–0.49)
	>65	4136	1734	0.42 (0.40–0.44)	0.30 (0.24–0.39)	985	0.24 (0.22–0.25)	0.37 (0.26–0.53)
Insulin	No	4605	2109	0.46 (0.44–0.48)	(reference)	933	0.20 (0.19–0.22)	(reference)
	Yes	338	159	0.47 (0.40–0.55)	1.03 (0.87–1.21)	247	0.73 (0.64–0.83)	3.60 (3.13–4.15)
Oral antidiabetic medication	No	4170	1929	0.46 (0.44–0.48)	(reference)	925	0.22 (0.21–0.24)	(reference)
	Yes	773	339	0.44 (0.39–0.49)	0.95 (0.84–1.06)	255	0.33 (0.29–0.37)	1.49 (1.29–1.71)
Simvastatin	No	3853	1826	0.47 (0.45–0.50)	(reference)	889	0.23 (0.22–0.25)	(reference)
	Yes	1090	442	0.41 (0.37–0.44)	0.86 (0.77–0.95)	291	0.27 (0.24–0.30)	1.16 (1.01–1.32)
Atorvastatin	No	4563	2086	0.46 (0.44–0.48)	(reference)	1061	0.23 (0.22–0.25)	(reference)
	Yes	380	182	0.48 (0.41–0.55)	1.05 (0.90–1.22)	119	0.31 (0.26–0.37)	1.35 (1.11–1.63)
Rosuvastatin	No	4825	2204	0.46 (0.44–0.48)	(reference)	1142	0.24 (0.22–0.25)	(reference)
	Yes	118	64	0.54 (0.42–0.69)	1.19 (0.92–1.52)	38	0.32 (0.23–0.44)	1.36 (0.98–1.88)
Ketorolac	No	4795	2228	0.46 (0.45–0.48)	(reference)	1172	0.24 (0.23–0.26)	(reference)
	Yes	148	40	0.27 (0.19–0.37)	0.58 (0.42–0.79)	8	0.05 (0.02–0.11)	0.22 (0.11–0.44)
Glaucoma	No	4508	2072	0.46 (0.44–0.48)	(reference)	1079	0.24 (0.23–0.25)	(reference)
	Yes	436	196	0.45 (0.39–0.52)	0.98 (0.85–1.13)	101	0.23 (0.19–0.28)	0.97 (0.79–1.19)
Connective tissue diseases	No	4818	2222	0.46 (0.44–0.48)	(reference)	1144	0.24 (0.22–0.25)	(reference)
	Yes	125	46	0.37 (0.27–0.49)	0.80 (0.60–1.07)	36	0.29 (0.20–0.40)	1.21 (0.87–1.69)
Chronic coronary heart disease	No	4481	2105	0.47 (0.45–0.49)	(reference)	1066	0.24 (0.22–0.25)	(reference)
	Yes	463	163	0.35 (0.30–0.41)	0.75 (0.64–0.88)	114	0.25 (0.20–0.30)	1.04 (0.85–1.26)

**Table 3:** Incidence rate ratios (with 95% confidence intervals) based on Poisson regression model. Adjusted for calendar year and time since start of follow-up.

	**Nd:YAG-laser treatment**	**Anti-VEGF Injection**
Sex (male vs female)		1.61 (1.47–1.77)	0.84 (0.75–0.94)
Age (yr)	<45	(reference)	(reference)
	45–65	0.44 (0.33–0.57)	0.46 (0.31–0.69)
	>65	0.30 (0.23–0.38)	0.55 (0.38–0.81)
Insulin	(yes vs no)	1.09 (0.92–1.29)	3.34 (2.86–3.90)
Oral antidiabetic medication	(yes vs no)	1.03 (0.91–1.16)	0.99 (0.85–1.16)
Simvastatin	(yes vs no)	0.93 (0.83–1.04)	1.10 (0.95–1.27)
Atorvastatin	(yes vs no)	1.15 (0.98–1.34)	1.11 (0.91–1.36)
Rosuvastatin	(yes vs no)	1.29 (1.00–1.66)	1.15 (0.82–1.60)
Other statin	(yes vs no)	1.03 (0.80–1.33)	0.69 (0.45–1.05)
Ketorolac	(yes vs no)	0.59 (0.43–0.81)	0.30 (0.15–0.60)
Diagnosis			
	H25.1	(reference)	(reference)
	H25.0	0.09 (0.05–0.16)	0.23 (0.13–0.42)
	H25.8	0.23 (0.18–0.30)	0.35 (0.26–0.47)
	H26.02	0.52 (0.38–0.72)	0.48 (0.27–0.84)
	Other	1.03 (0.85–1.25)	2.04 (1.66–2.51)
Glaucoma	(yes vs no)	0.97 (0.84–1.12)	0.91 (0.74–1.12)
Connective tissue diseases	(yes vs no)	0.75 (0.56–1.00)	1.13 (0.81–1.58)
Chronic coronary heart disease	(yes vs no)	0.85 (0.72–1.00)	0.86 (0.70–1.05)

Anti-VEGF, anti-vascular endothelial growth factor; Nd:YAG, neodymium-doped yttrium aluminum laser

##  RESULTS

Altogether 269,929 cataract operations took place in Finland between July 1, 2010 and December 31, 2016. In 135,305 (50.1%) of the operations, the laterality of the cataract surgery was not known, so these operations were excluded. Finally, our study population consisted of 89,201 individuals who underwent cataract surgery, with 89,201 right eyes and 86,851 left eyes in our cohort (*n* = 176,052 operations).

The baseline characteristics of the study's patients are shown in Table 1. The cataract operations were performed in 11 hospital districts representing both high- and low-volume clinics around Finland whose population is 5.5 million. In our study, the majority of the eyes (93.7%) were operated due to normal age-related cataract (coded as H25.0, H25.1, H25.8), 2.1% of operated eyes had pre-senile cataract, and 4.2% were operated due to trauma or other eye disease.

Of the operated patients, 62.7% were female. The median age of the patients was 75.6 years (range 68.9–81.1). Of note, of all operated patients, 35.2% used statin therapy (simvastatin 21%, atorvastatin 8.7%, rosuvastatin 2.8%, other statin 2.7%). The proportion of statin users differed among specific cataract subgroups, the lowest being among patients who were operated on due to pre-senile cataract (coded as H26.02; 17.9%).

As regards to the systemic comorbidities, 14.3% had diabetes, 12.4% systemic hypertension, 10% chronic heart disease, 2.7% rheumatoid arthritis, 1.9% hypothyrosis, 1.8 % prostate cancer, and 1.6% had breast cancer. According to our analysis, these comorbidities did not reveal any associations with main event rates. Altogether, 8.9% of all cataract operations were performed on glaucoma patients.

After cataract surgery, the intravitreal anti-VEGF injection was administered to 1180 (0.7%) operated eyes, of which 191 eyes were diagnosed with diabetic macular edema (DME) and 989 eyes with CME. Nd:YAG-laser capsulotomy was performed due to the development of PCO on 2268 (1.3%) eyes.

Combinatory topical antibiotic-cortisone eye drops were used in the majority of the operated study eyes. Topical NSAID (ketorolac) was used in 3460 (2.0%) of the operated eyes, the majority of the NSAID (ketorolac) users being in the H25.8 group (5.5%).

### The Incidence Rates of Secondary Surgery

Overall incidence rates (any event per 100 person-years) showed that out of all the cataract-operated patients, according to the univariate model, those with insulin treatment had an 83% higher risk for secondary operations (intravitreal anti-VEGF injection or Nd:YAG-laser) (IRR1.21, 95% confidence interval, CI: 1.10–1.34), however, only 13% of diabetic patients with OAD had a higher risk for secondary operations (intravitreal anti-VEGF injection or Nd:YAG-laser). Of note, female patients had a 20% higher risk for both intravitreal injection or Nd:YAG-laser operations (IRR 1.20, 95% 1.12–1.29) [Supplemental Table 1].

When intravitreal anti-VEGF injections and Nd:YAG-laser were studied separately, significant differences were observed [Table 2]. In males, the incidence of Nd:YAG-laser was higher than in females, but the association was the opposite for intravitreal anti-VEGF injections. The incidence for intravitreal anti-VEGF injections was higher in insulin users (IRR 3.60, 3.13–4.15). Cumulative events curves showed that during the first two postoperative years, the probability of administering intravitreal anti-VEGF injections was more common than the probability of using Nd:YAG- laser, however, after two years the situation was reversed [Figure 1].

### Poisson Regression Analysis

According to our analysis, after adjusting for age and chronic diseases, we detected that insulin usage among cataract-operated patients was associated with a higher risk for intravitreal anti-VEGF injection (IRR 3.5, 95% CI: 2.9–4.1; *P* < 0.0001) treatment, but the use of OAD was not (IRR 1.1, 95% 0.92–1.33, *P* = 0.30).

The unadjusted incidence rates showed that systemic use of simvastatin (IRR 1.16, 95% CI: 1.0–1.32), atorvastatin (IRR 1.35, 95% CI: 1.1–1.6), and rosuvastatin (IRR 1.36, 95% CI: 0.98–1.9) were associated with an increased rate of intravitreal injections after cataract surgery [Table 2]. However, after an adjustment for baseline confounders with the Poisson model, no differences were found.

We found no association between the usage of rosuvastatin (*P* = 0.05), simvastatin (*P* = 0.21), or atorvastatin (*P* = 0.09) and the incidence rate for Nd:YAG-laser treatment in our cohort.

Of note, the use of topical NSAID (ketorolac) was associated with reduced intravitreal injection rates (IRR 0.3; 95% CI: 0.15–0.60, *P* = 0.0007) as well as reduced incidence of Nd:YAG-laser (IRR 0.59; 95% CI: 0.43–0.81, *P* = 0.0011) treatments after cataract operations.

Accordingly, the female gender was associated with a decreased rate of intravitreal injections (IRR 0.84; 95% CI: 0.74–0.94, *P* = 0.0026) administration, but with a significantly increased rate of Nd:YAG-laser treatments (IRR 1.67; 95% CI: 1.47–1.78, *P* < 0.0001).

### Predicted Incidence Curves of Intravitreal Injections and Nd:YAG Laser Procedures After Cataract Surgery

Predicted incidence plots showed that the incidence of Nd:YAG-laser capsulotomy peaked about four-and-a-half years after cataract operation [Figure 2]. Conversely, intravitreal injections were given typically during the first few months after surgery or later after about four-and-a-half years after surgery.

##  DISCUSSION

In our study, based on 176,052 cataract operations performed during the years 2010 to 2016 in Finland, the use of topical NSAID (ketorolac) was associated with a 70% (40–85%) reduced incidence rate of intravitreal anti-VEGF injection to treat postoperative ME and a 41% (19–57%) reduced incidence rate of Nd:YAG-laser to treat PCO or fibrosis after cataract surgery. Unlike ketorolac, systemic statin use was not associated with the rate of Nd:YAG-laser capsulotomy after cataract surgery. Although there was a trend toward a statistically significant association between systemic rosuvastatin use and an increased need for Nd:YAG-laser capsulotomy, this finding could still be a statistical coincidence, as no association was found between simvastatin or atorvastatin usage and the incidence rate of Nd:YAG laser treatment.

Despite all technological advancements, cataract surgery always induces an inflammatory response, leading to side effects, CME and PCO. ${}^{\left[4,15,16\right]}$ Today, NSAIDs are used to treat the postoperative anterior chamber inflammation after cataract surgery either combined with steroids or alone. ${}^{\left[17,18\right]}$ However, topical medication protocols vary worldwide, being specific to each surgical unit. Of note, in our register-based study, the operated eyes were routinely treated with combined antibiotic-steroid eye drops, and only minority (2%) of eyes received topical NSAID (ketorolac), confirming our recent findings that revealed that the least common combination after cataract operation was the triple treatment (steroids, antibiotic, and topical NSAID).^[19]^ Out of other topical commercially available NSAIDs (nepafenac, bromfenac, diclofenac), only diclofenac was used in our study, but could not be analyzed due to the limited number of eyes treated.

Historically, the treatment protocol with topical NSAIDs after cataract surgery began in the 1970s. ${}^{\left[20,21\right]}$ In the first double-blind, randomized, single-center study of 59 adults undergoing cataract surgery, topical ketorolac tromethamine 0.5% was shown to be as effective and well-tolerated as prednisolone acetate 1% solution in controlling postoperative inflammation and pain.^[22]^ The addition of topical bromfenac to steroids was shown to reduce inflammation better than the use of steroids alone after cataract surgery.^[23]^ Pretreatment, by NSAIDS or steroids, has been shown to reduce postoperative inflammation and risk of CME.^[24]^Recently, topical NSAIDs offered efficacy comparable to steroids in reducing postoperative inflammation, however, NSAIDs were also superior in reducing the risk of CME after cataract surgery.^[3]^

Of note, in our study, the use of systemic statin was neither beneficial nor detrimental after cataract surgery. The pathogenesis of PCO is known to include a fibrotic reaction of lens epithelial cells caused by transforming growth factors, and statins are known to prevent fibrosis.^[8,25]^ In Finland we have official guidelines to treat secondary cataract once the visual acuity has dropped to < 0.6 on Snellen, meaning that we do not treat the secondary cataract patients with VA better than 0.6 in the tertiary clinics. However, in the private sector there are no such strict guidelines. Despite many reports suggesting that statins could reduce ﬁbrosis in various ophthalmic conditions,^[7,10,26]^no association was found in the incidence rate of Nd:YAG capsulotomies in statin-treated cataract patients as compared with non-statin treated.

We do acknowledge the following limitations. Our study was based on administrative register data, and there are some inaccuracies in procedure coding such as missing data. We considered only baseline medications (at the time when the cataract operation took place). Therefore, it is probable that, for example, statin usage was changed during the six-year follow-up period. It is also worth mentioning that the prevalence of postoperative macular edema (ME) is known to vary from study to study depending on how ME is defined. Previous studies have shown that there is a low incidence of 0.1% to 2.4% of clinically significant postoperative ME in patients with no risk factors. In our study, altogether, 0.7% of study eyes received anti-VEGF injections for postoperative ME after cataract surgery. In Finland, in cases with postoperative ME (either CME or DME), there is a recommendation to use topical NSAID for two months postoperatively. If there is no response to topical NSAID, the patient will then be referred to a Medical Retina unit for consultation including Optical Coherence Tomography measurements, initiation of anti-VEGF treatment, and in very rare cases for insertion of a dexamethasone implant. In addition to diabetes, many ophthalmic factors are also related to the increased risk of ME. These include the duration of phaco surgery, the need for additional instrumentation due to small pupils or exfoliation syndrome (including iris hooks or Malyugin ring), intraoperative complications, and the severity profile of the operated eyes (dense cataract). Unfortunately, these important factors could not be taken into account in this observational register-based study.

Cataract causes 25% of global blindness, especially in the developing world.^[28]^ Compared with a recent paper, cataract patients in our study were older and the proportion of patients with comorbidities such as diabetes and hypertension was smaller.^[29]^ In Finland with a care guarantee, cataract surgery is available for everybody in need, despite household income status, education, or occupation. In Finland, the cataract surgery rate is 7000 per 1 million people, which is proportionate to the range of 4000 to 10,000 per million performed in developed countries.^[30]^

In conclusion, our study is the first population-based study of cataract-operated eyes providing an estimate of anti-VEGF and Nd:YAG laser procedures in Finland post cataract surgery. Based on our register study, we recommend use of topical non-steroidal anti-inflammatory prophylaxis after cataract surgery, unless the patient has systemic contraindications such as asthma, because topical NSAID (ketorolac) was associated with a remarkably lower incidence rate of Nd:YAG-laser and intravitreal anti-VEGF injection after cataract surgery. However, although systemic statin use did not seem to have beneficial effects after cataract surgery as regards the development of PCO or CME, it was good to observe that neither did it seem to increase the burden of ophthalmic interventions after cataract surgery.

##  Financial Support and Sponsorship

This study was supported by Y1014SILM1 grant (SL) and funded by University of Helsinki, Finland (JH).

##  Conflicts of Interest

There are no proprietary interests or conflicts of interest related to this submission.
